# Patient Perceptions on Data Sharing and Applying Artificial Intelligence to Health Care Data: Cross-sectional Survey

**DOI:** 10.2196/26162

**Published:** 2021-08-26

**Authors:** Ravi Aggarwal, Soma Farag, Guy Martin, Hutan Ashrafian, Ara Darzi

**Affiliations:** 1 Institute of Global Health Innovation Imperial College London London United Kingdom

**Keywords:** artificial intelligence, patient perception, data sharing, health data, privacy

## Abstract

**Background:**

Considerable research is being conducted as to how artificial intelligence (AI) can be effectively applied to health care. However, for the successful implementation of AI, large amounts of health data are required for training and testing algorithms. As such, there is a need to understand the perspectives and viewpoints of patients regarding the use of their health data in AI research.

**Objective:**

We surveyed a large sample of patients for identifying current awareness regarding health data research, and for obtaining their opinions and views on data sharing for AI research purposes, and on the use of AI technology on health care data.

**Methods:**

A cross-sectional survey with patients was conducted at a large multisite teaching hospital in the United Kingdom. Data were collected on patient and public views about sharing health data for research and the use of AI on health data.

**Results:**

A total of 408 participants completed the survey. The respondents had generally low levels of prior knowledge about AI. Most were comfortable with sharing health data with the National Health Service (NHS) (318/408, 77.9%) or universities (268/408, 65.7%), but far fewer with commercial organizations such as technology companies (108/408, 26.4%). The majority endorsed AI research on health care data (357/408, 87.4%) and health care imaging (353/408, 86.4%) in a university setting, provided that concerns about privacy, reidentification of anonymized health care data, and consent processes were addressed.

**Conclusions:**

There were significant variations in the patient perceptions, levels of support, and understanding of health data research and AI. Greater public engagement levels and debates are necessary to ensure the acceptability of AI research and its successful integration into clinical practice in future.

## Introduction

Recent advances in data science and artificial intelligence (AI) technologies have the potential to transform the way patient-centered health care is delivered [1]. AI is a branch of computer science that refers to the ability of computers or machines to creatively solve problems that would normally require human intelligence. Machine learning (ML) is a subset of AI that provides systems with the ability to automatically learn and improve from experience without explicitly being programmed. It involves algorithms that are designed to emulate human intelligence by learning from their environment [2]. Considerable research is being conducted as to how AI and ML can be applied to health care, with diagnostics seeming to be the most promising field for AI implementation [3,4]. However, for AI research to be successful and truly translational, large amounts of health data are required for training and testing algorithms [5]. Therefore, public trust and support for using health data in AI research are essential.

Public perceptions regarding sharing of health data for research are well characterized [6-8]. Although concerns regarding the privacy, confidentiality, and commercial motives associated with data sharing are frequently highlighted, when people perceive that public or societal benefits arise from such research and when they place trust in the organizations conducting the research, they are generally supportive [7]. However, patient and public perceptions regarding health data sharing for AI research are not sufficiently characterized [9]. Data sharing for AI research purposes is a controversial subject, and therefore, conditional public support for data sharing cannot be assumed to extend to this field of research [10]. Reasons for this include knowledge and understanding of AI in general [10], ethical concerns [11], and fears around the potential reidentification of anonymized personal health data [12]. Furthermore, recent negative media reports about large technology companies using health data for AI research [13] and several important data breaches and cyberattacks [14] may undermine public trust in this technology.

Despite these additional issues, there is limited research exploring patient perceptions on data sharing for AI research purposes [10,15-18]. If the promises of AI are to be truly realized in health care, strategic public debates are important to ensure that the public maintains trust in the technology and use of confidential health data [19]. This is now especially important as regulatory approval has already been granted for AI-powered diagnostic software to be used in routine clinical practice [20].

Therefore, the aim of this study was to survey a large sample of patients at our hospital to identify their current awareness on health data research, and viewpoints on data sharing for AI research purposes and using AI technology on health care data.

## Methods

### Survey Development

We conducted a cross-sectional study using a self-completed questionnaire survey tool with patients at a large, multisite university teaching hospital in London. The survey tool was developed via a multistep codesign process in collaboration with patients. First, a literature review was conducted to identify the initial survey themes and items, which were then used to inform the codesigning process of a prototype questionnaire with a patient focus workshop. The workshop was a 3-hour face-to-face meeting with subject matter experts and a group of 3 patients selectively chosen out of 9 individuals who applied. The patients were chosen for their experience in survey development and had previously been involved in research studies at our organization. The feedback and suggestions from the workshop were analyzed by two researchers (RA and HA) and changes were made to the prototype questionnaire based on this feedback. The revised survey was then emailed to the workshop participants for further review with no more changes suggested. Finally, a pilot study was conducted with 5 patients of varying ages, genders, education levels, and ethnicities recruited opportunistically from an outpatient clinic in our hospital to evaluate comprehension and measure the average time taken to complete the survey. We were able to ascertain that all patients understood the information sheet and the questions, and they were able to complete the survey within 12 minutes.

### Sample

The participants were opportunistically recruited from outpatient waiting areas or from the inpatient wards over a 12-week period beginning June 2018. The eligibility criteria for participation were as follows: (1) 16 years or older, (2) able to understand the information describing the research study, and (3) willing and able to provide informed written consent. The study was reviewed and approved by the South East Scotland Research Ethics Service (18/SS/0057/AM01).

Data were collected on patient and public views about sharing health data for research and the use of AI on health data. The front page of the questionnaire introduced the participants to AI, electronic health records, and data anonymization and sharing. The participants were informed about the aims of the questionnaire, and they voluntarily participated after being given a patient information sheet and the opportunity to ask questions. Patient anonymity was ensured, and the responses were identified by participant identification numbers only. The 24-item questionnaire examined various aspects related to patient and public views on the subject and was split into 4 sections:

1. awareness of health data usage for research
2. views on data sharing, consent, and anonymization
3. views on AI
4. sociodemographic characteristics and health statuses of the participants

### Statistical Analysis

All the surveys were completed on paper before being manually entered into a database in Microsoft Excel (Microsoft Corporation). Descriptive statistics were used to describe the sample by gender, age, ethnicity, educational attainment, perceived health status, Internet usage, and smartphone ownership. The age categories included 16-30, 31-45, 46-64, and 65+. Educational attainments were classified as “low” (General Certificate of Secondary Education [GCSE] or below), “medium” (Advanced Certificate of Secondary Education [A-Level] or equivalent) or “high” (university degree and above). Ethnicities were grouped as either “Caucasian” (White/British or White/Other) or “Black, Asian, and minority ethnic (BAME)” (African/Caribbean, Asian, mixed or multiple ethnicities, or other). Personal health statuses were classified as “high” (good, very good, or excellent) or “low” (poor or fair). Internet usage was categorized as “daily” or “less frequent/no access” and smartphone ownership as “yes” or “no/prefer not to say.”

For questions with Likert-type ordinal responses, ordinal logistic regression was performed to examine the relationships between the responses and the demographic variables mentioned above. Binary logistic regression was used for questions with binary responses. These methods were used because of the nature of the dependent and independent variables and because they could be adjusted for other demographic variables, and any confounding effects could be removed. For each demographic variable, the categories were compared with a predefined reference group for performing logistic regression. The reference groups were “female” for the sex variable, 65+ for age, BAME for ethnicity, “high” for education level, “low” for personal health status, “less frequent/no access” for Internet usage, and “no” for smartphone ownership. The results were deemed statistically significant if *P*<.05. Statistical analysis was performed using SPSS (version 27.0, IBM Corp).

### Data Sharing

Access to deidentified data might be provided on reasonable request when accompanied by a study protocol and analysis plan. Requests are subject to the establishment of appropriate data governance and approval by a committee involving the current research team. Requests must be made in writing to the corresponding author.

## Results

### Participants

A total of 408 participants recruited from all 5 sites of a multicenter university teaching hospital in the United Kingdom completed the survey. The demographic characteristics of the respondents are presented in Table 1. Internet usage (59/60, 98.9% in the 16-30 group mentioning daily usage compared to 48/61, 78.9% in the 65+ group) and smartphone ownership (59/60, 98.9% in the 16-30 group compared to 35/61, 57.9% in the 65+ group) declined with increasing age. Daily Internet usage reduced with reducing educational attainments (158/167, 94.6% in the “high” group [university degree and above] compared to 56/67, 83.1% in the “low” group [GCSE and below]). Similarly, smartphone use decreased with decreasing educational attainment (158/167, 94.6% in the “high” group compared with 48/67, 71.5% in the “low” group). Moreover, 90.9% (286/315) of smartphone users used the Internet daily compared to 75.5% (40/53) of non-smartphone Internet users.

**Figure 1:**
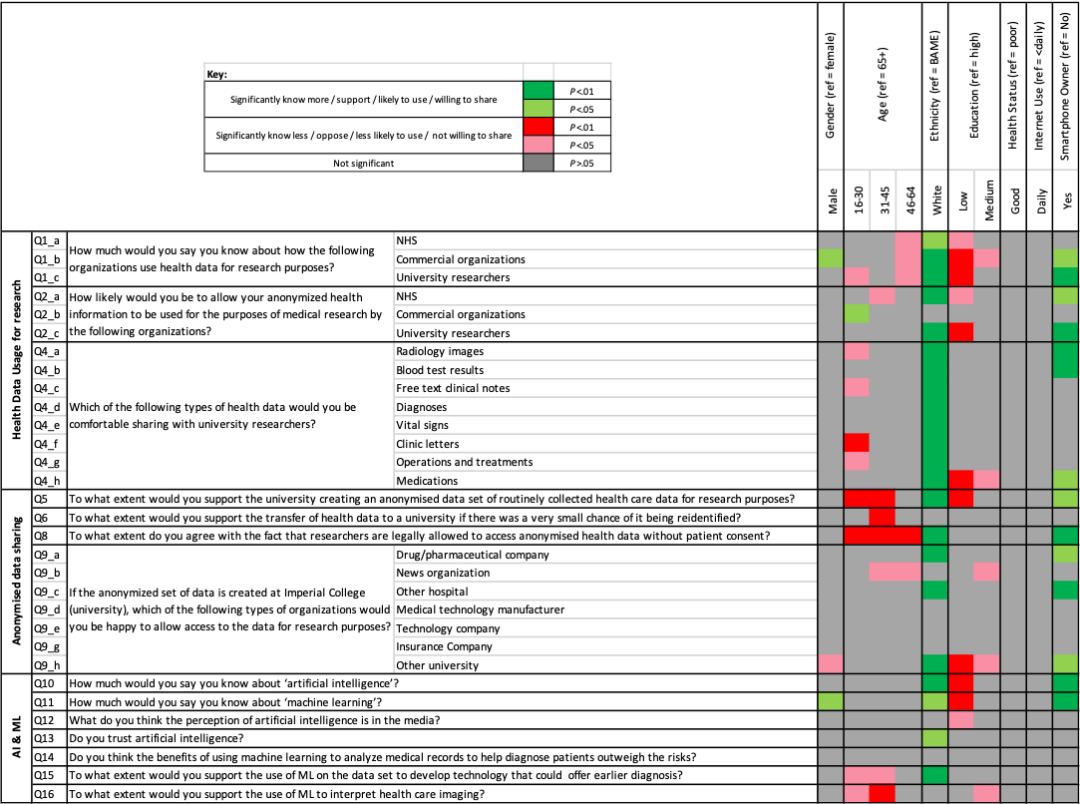
Significance map detailing the directionality and significance of the relationships between the responses and the panel of demographic characteristics. BAME: Black, Asian, and minority ethnic; ML: machine learning; NHS: National Health Service.

The full breakdown of the questions and answers are given in Tables 2 and 3. Figure 1 shows a significance map with details on the directionality and level of significance associated with the responses and all the demographic variables (see Multimedia Appendix 1 for the results of the logistic regression analyses).

**Table 1:** Demographic characteristics of the respondents.

Characteristic			Number of respondents (N=408)		Percentage (%)
**Gender**					
	Male	173		42.4	
	Female	198		48.5	
	Unanswered	37		9.1	
**Age (years)**					
	16-30	90		22.1	
	31-45	81		19.9	
	46-64	123		30.1	
	65-79	61		15	
	>80	15		3.7	
	Unanswered	38		9.3	
**Ethnicity**					
	White/British	174		42.6	
	White/Other	55		13.5	
	African/Caribbean	45		11	
	Asian	56		13.7	
	Mixed or multiple ethnic	10		2.5	
	Other	26		6.4	
	Unanswered	42		10.3	
**Education**					
	No qualifications	34		8.3	
	GCSE^a^/O-Level^b^/NVQ^c^	67		16.4	
	A-Level^d^	70		17.2	
	University degree	167		40.9	
	Other	29		7.1	
	Unanswered	41		10.0	
**Personal health status**					
	Poor	42		10.3	
	Fair	107		26.2	
	Good	119		29.2	
	Very good	86		21.1	
	Excellent	18		4.4	
	Unanswered	36		8.8	
**Internet usage**					
	Daily	301		73.8	
	Less frequent	42		10.3	
	No access	29		7.1	
	Unanswered	36		8.8	
**Smartphone ownership**					
	Yes	315		77.2	
	No	53		13.0	
	Prefer not to say	4		1.0	
	Unanswered	36		8.8	

^a^GCSE: General Certificate of Secondary Education.

^b^O-Level: General Certificate of Education Ordinary Level

^b^NVQ: National Vocational Qualifications.

^c^A-Level: General Certificate of Education Advanced Level.

**Table 2:** Respondents’ opinions on health data usage and sharing for research.

Question			Number of respondents		Responses								
**1. How much would you say you know about how the following organizations use health data for research purposes?**					Never heard of (%)		Heard of, know nothing about (%)		Just a little (%)		A fair amount (%)		A great deal (%)
	a. NHS^a^	407		14.3		13.3		30.7		26.8		15	
	b. Commercial organizations	405		25.7		22.7		27.9		15.1		8.6	
	c. University researchers	405		18.5		21.7		32.1		19.5		8.1	
**2. How likely would you be to allow your anonymized health information to be used for the purposes of medical research by the following organizations?**					Very unlikely (%)		Fairly unlikely (%)		Not sure (%)		Fairly likely (%)		Very likely (%)
	a. NHS	408		4.4		3.9		13.7		30.4		47.5	
	b. Commercial organizations	405		22.0		17.5		34.1		14.1		12.3	
	c. University researchers	405		6.7		6.4		21.2		31.4		34.3	
					Very unlikely (%)		Fairly unlikely (%)		Not sure (%)		Fairly likely (%)		Very likely (%)
3. To what extent would you support ICL^b^ creating a large, anonymized set of data of routinely collected ICHNT^c^ health care data for AI^d^ research purposes?			408		1.5		2.5		12.3		48.5		35.3
					Very unlikely (%)		Fairly unlikely (%)		Not sure (%)		Fairly likely (%)		Very likely (%)
4. To what extent would you support the transfer of your anonymized health data to ICL if there was a very small chance of it being reidentified after transfer?			407		13.0		13.5		17.2		36.3		19.9
					Strongly disagree (%)		Disagree (%)		Neither agree nor disagreed (%)		Agree (%)		Strongly agree (%)
5. Currently, researchers are legally allowed to access anonymized health data for research without the need for patient consent. To what extent do you agree with this?			407		9.3		14.7		20.1		39.1		16.7

^a^NHS: National Health Service.

^b^ICL: Imperial College London

^c^ICHNT: Imperial College Healthcare NHS Trust.

^d^AI: artificial intelligence.

**Table 3:** Respondents’ opinions on artificial intelligence and machine learning.

Question	Number of respondents	Responses				
		Never heard of (%)	Slightly aware (%)	Somewhat aware (%)	Moderately aware (%)	Extremely aware (%)
How much would you say you know about "artificial intelligence?"	407	22.9	20.4	20.9	29.2	6.6
		Never heard of (%)	Slightly aware (%)	Somewhat aware (%)	Moderately aware (%)	Extremely aware (%)
How much would you say you know about “machine learning?”	407	27.3	21.9	22.4	22.9	5.7
		Very negative (%)	Slightly negative (%)	Not sure (%)	Slightly positive (%)	Very positive (%)
What do you think the perception of artificial intelligence is in the media?	207	1.4	29.5	25.6	34.3	9.2
		Strongly distrust (%)	Slightly distrust (%)	Not sure (%)	Slightly trust (%)	Strongly trust (%)
Do you trust artificial intelligence?	206	3.4	12.6	29.5	43.5	10.6
		Risk outweighs benefits (%)	Risk and benefits equal (%)	Benefits outweighs risk (%)	Don’t know (%)	
Do you think the benefits of using machine learning to analyze medical records to help diagnose patients outweighs the risks?	205	6.8	22.7	45.9	23.7	
		Strongly oppose (%)	Tend to oppose (%)	Neither support nor oppose (%)	Tend to support (%)	Strongly support (%)
To what extent would you support the use of machine learning to develop technology that could potentially offer earlier diagnosis and more accurate treatments to patients?	206	0.5	3.4	8.7	52.7	34.3
		Strongly oppose (%)	Tend to oppose (%)	Neither support nor oppose (%)	Tend to support (%)	Strongly support (%)
To what extent would you support the use of machine learning to interpret health care imaging as an aid for doctors when reporting these images?	206	1.0	2.9	9.7	48.1	38.3

### Awareness of Health Data Usage for Research

#### NHS

Among the 407 respondents, 170 (41.7%) knew “a fair amount’” or “a great deal” about how the NHS uses health data for research purposes (Question 1a), and 318/408 (77.9%) were “fairly likely” or “very likely” to allow their anonymized health information to be used for medical research purposes by the NHS (Question 2a). In comparison with their reference group, those aged 31-45 (*P*=.013) and with lower educational attainment (*P*=.019) were significantly less likely to be comfortable sharing health data, whereas Caucasian groups (*P*<.001) and those who own smartphones (*P*=.014) were more likely to be comfortable sharing data with the NHS for research purposes.

#### Commercial Organizations

Only 96/405 (23.7%) knew “a fair amount” or “a great deal” about how commercial organizations use health data for research purposes (Question 1b), and 107/405 (26.4%) were “fairly likely” or “very likely” to allow their anonymized health information to be used for medical research purposes by commercial organizations (Question 2b). In comparison with their reference group, those aged 16-30 (*P*=.042) were significantly more likely to be comfortable sharing data with commercial organizations for research purposes.

#### University Researchers

Of the 405 respondents, 112 (27.7%) knew “a fair amount” or “a great deal” about how university researchers use health data for research purposes (Question 1c), and 266/405 (65.7%) were “fairly likely” or “very likely” to allow their anonymized health information to be used for medical research purposes by university researchers (Question 2c). In comparison with their reference group, those of lower educational attainment (*P*=.003) were significantly less likely to be comfortable sharing health data, whereas Caucasian groups (*P*<.001) and those owning smartphones (*P*=.007) were more likely to share data with university researchers.

As for the types of data shared with university researchers, over 70% of respondents were comfortable sharing information on radiology, blood test results, diagnoses, operations and treatments and medications (Question 4). However, fewer respondents were comfortable sharing clinic letters (51%), free text clinical notes (51.2%), or vital signs (67.2%). Caucasian respondents were significantly more likely to be comfortable sharing all data types (*P*=.001). Those under 30 were less likely to be comfortable sharing data on operations and treatments, free text clinical notes, and radiology images (all *P*<.05), and clinic letters (all *P*<.01). Smartphone owners were more likely to be comfortable sharing radiology images, blood test results (all *P*<.01), and medication data (all *P*<.05).

### Data Sharing, Consent, and Anonymization

Among the 408 respondents, 342 (83.8%) “tend to support” or “strongly support” the creation of an anonymized data set of routinely collected NHS data for AI research purposes at the university partner (Question 5). In comparison to their reference counterpart, respondents under the age of 45 (*P*=.002) or having lower educational achievement (*P*=.003) were statistically less likely to support data set creation, whereas those of Caucasian background (*P*=.006) and smartphone owners (*P*=.033) were more likely to support this. Fewer respondents would support the transfer of anonymized routinely collected health data to a university partner if there was a small chance of reidentification after transfer (229/407, 56.2%) (Question 6). Those aged 31-45 were significantly less likely to support this when compared with the reference group (*P*=.008).

Furthermore, greater than 50% (227/407, 55.7%) of the respondents cited that individual-level patient consent should not be required to use anonymized routinely collected health care data for research purposes, as is the status quo (Question 8). All age groups below 65 were significantly less likely to agree with this compared with those over 65 (all *P*<.01). Those of Caucasian background (*P*<.001) and smartphone owners (*P*=.008) were more likely to agree.

With respect to allowing third party organizations access to anonymized data for research purposes, respondents were uncomfortable sharing data with news organizations (6.9%), insurance companies (6.9%), and technology companies (21.6%) (Question 9). Those aged 31-64 and with medium educational attainment were significantly less inclined to provide access to news organizations (all *P*<.05). Respondents were slightly more inclined to provide data access to drug/pharmaceutical companies (47.1%), medical technology companies (46.1%), other universities (44.1%), and other hospitals (68.9%). Caucasians were significantly more comfortable with providing access to these organizations. Females and those of low and medium educational attainments were significantly less likely to be comfortable sharing data with other universities.

#### AI and ML Research

More respondents were familiar with AI (231/407, 56.7%) than ML (207/407, 50.8%). Further, 22.9% (93/407) and 27.3% (111/407) had never heard of AI and ML, respectively. Patients from Caucasian backgrounds (*P*=.003, *P*=.028), males (*P*=.066, *P*=.025), and smartphone owners (*P*<.001, *P*<.001) were significantly more aware about AI and ML in comparison with their reference groups (Question 10). Those of lower educational attainment are significantly less familiar with these terminologies (*P*<.001, *P*<.001).

As we identified that 49.2% (200/407) of respondents stated they had “not heard of” or were only “slightly aware” of ML, the responses from those respondents were excluded from the results of questions 12-16. Moreover, 90/207 (43.5%) think that the perception of AI in the media is very positive or slightly positive and 112/206 (54.1%) of respondents strongly trust or slightly trust AI. Caucasians have significantly more trust in AI (*P*=.035) than BAME patients. Furthermore, 95/205 (45.9%) think that the benefits of AI in health care outweighed the risks compared with 6.8% (14/205) who think that the risks outweigh the benefits (Question 14). With regard to supporting ML research, 87.4% (180/206) and 86.4% (178/206) strongly support or tend to support this on anonymized health care data and health care imaging respectively (Questions 15 and 16). Caucasians were significantly more likely to support this research (*P*=.01), whereas those aged 16-30 and 31-45 were significantly less supportive of this research on health care data (*P*=.013 and *P*=.027 respectively).

## Discussion

### Major Findings

The increasing availability of health care data and exponential rise of computational power have caused the recent surge in AI applications in health care [5]. Powerful AI techniques can potentially assist physicians to make better clinical decisions or even perform some tasks autonomously. The successful integration and translation of this technology into routine clinical practice, depends not only on numerous technological challenges, but also whether the public and patients can accept and trust it [21].

In this study, which to the best of our knowledge is the first one assessing patients views about sharing health care data for AI research from a UK hospital, several key findings emerged. Consistent with previous literature [10], we found that patients report generally low levels of knowledge about AI and ML. This is a key finding; if the use of AI in healthcare is to increase, educating patients about the risks and benefits of this technology is crucial [19]. The vision of AI presented in the press and other forms of media [22] can be very different from reality; as such, engagement and education from trusted sources [19,23] or using realistic AI-based health scenarios [10] are required. This lack of knowledge may also be problematic when considering the process of informed consent for any future AI interventions [24]. Despite this challenge, we identified that patients were generally more trusting of AI than not and a large proportion thought that the benefits outweighed the potential risks.

Patients report that they are more knowledgeable about how the health service in the UK (NHS) uses health data for research than commercial organizations or university researchers. However, most patients would be comfortable sharing anonymized health data with the NHS and university researchers. Both are public institutions, and therefore, this demonstrates the importance of trust when sharing sensitive information. We also identified that patients were less willing to share data with commercial organizations. Privacy fears [7] and anxiety that the transferred data may be used for profit could explain this finding. This was especially the case with news organizations, technology companies, and insurance companies. Our findings add to a downward trend in public trust regarding sharing data with commercial organizations [25], which seems to have changed significantly when compared to historical evidence [26]. This suggests that recent technology scandals such as Cambridge Analytica [27] and media reports of inappropriate sharing of patient data with technology companies [13] have increased public awareness about the potential risks and consequences of data sharing with commercial companies [28]. Governmental guidelines and regulations [29,30] have recently been published to reassure patients that data-driven technology is safe and can maintain privacy, and they provide evidence of what good practice looks like to the industry and commissioners. These findings are similar to a recent systematic review [7], where the conditional nature of support for data sharing was identified. A variety of concerns including data security, privacy, anonymization, and control of data were also raised in this review.

Anonymization of data sets through deidentification is crucial to allow safe storage and sharing of health data while preserving privacy [7]. However, current processes for de identification have proved susceptible to reidentification attacks and the risk of this happening can never be completely eliminated [12]. There is also concerning evidence that even accepted deidentification techniques may not be sufficient to ensure privacy in the face of sophisticated AI algorithms [7]. This is especially concerning as AI research in health care requires large, granular data sets containing sensitive information, which if compromised could cause psychological and reputational harm to patients. Our study demonstrates that patients would be less supportive of data sharing if there was a probability of reidentification. In an attempt to mitigate this concern, the Information Commissioner’s Office (ICO), the United Kingdom’s independent statutory body for information rights, has issued a code of practice on anonymization [31]. In the United States, the Privacy Rules of the Health Insurance Portability and Accountability Act (HIPAA) provides similar guidance [32]. These guidelines, along with the introduction of the General Data Protection Regulations (GDPR) in Europe and enhanced cybersecurity [33], may allay public fears about reidentification of health data. However, despite these regulations, multiple privacy challenges specific to AI remain and updated ethical and legal frameworks are required to regulate the use of AI in health care [34].

Multivariate analysis revealed some differences in views across participant subgroups. Consistent with previous literature, BAME populations were generally less supportive of data sharing and AI research [10] along with younger age groups and those with lower educational attainment. Training and testing of AI algorithms require diverse data sets that are representative of the local population for which the algorithm will be deployed [35]. The lack of inclusion of minorities in AI data sets has been shown to induce algorithmic bias [36]. Educating BAME communities about the benefits of data sharing is required to help minimize this bias and ensure that AI research is representative of the target population. The differences noted across age groups may be related to the fact that older people may pay more attention to health and medical issues than younger people. There are opportunities to better engage younger people with creative approaches such as through social media, and these should be explored further [37,38].

Notwithstanding the issues outlined above, the majority of respondents in our study who had prior knowledge of AI would support AI research on health care data and imaging in a university setting. However, it is imperative to understand which health data are considered acceptable and unacceptable for AI research by patients. The authors believe that it is important that patients are not simply informed about how health data is used in AI research but are actively involved and consulted with in all aspects of the work. The involvement and guidance of patients and the public will ensure that using AI in health care is transparent, trustworthy, ethical, and socially beneficial.

### Limitations

Our results should be interpreted in the context of the limitations related to our study design. This was a cross-sectional questionnaire study that provides a snapshot of patients’ views and thoughts, rather than how these may change over time. This is particularly relevant to this study where data was collected 3 years ago because AI research is a rapidly advancing field with an abundance of new research and media articles published regularly. Therefore, it is inevitable that patients’ knowledge and viewpoints will change over time. The demographic characteristics of our patients and the fact that patients were recruited from only a UK public hospital may limit the generalizability of the findings. Furthermore, the convenience sampling technique used to approach patients for inclusion in this study signifies that the findings are not likely be generalizable to a wider population that may have no relationship with health services. Cross-sectional studies are also prone to nonresponse bias, which can result in a nonrepresentative sample. Unfortunately, the number of patients who declined to complete the questionnaire was not accurately measured in this study (although approximately 1000 patients were approached); hence, it is difficult to measure the effect of this aspect. There is a risk of selection biases caused by the survey being in English, but we attempted to minimize selection bias by recruiting patients on different days and times and from different areas of the hospitals. Although definitions and clarifications about AI and health data research were provided and we conducted pilot work to simplify the questions, the survey concepts were complex; therefore, some respondents may have not fully understood the information provided.

### Conclusions

With increasing research on implementing AI in health care, more attention is given to the public opinion and acceptability of this type of research on health data. This study has demonstrated that there are significant variations in the patients’ perception, knowledge and understanding of health data research and AI. There is a need for greater awareness among the public and patients, which can only be achieved by public engagement and debates. This will be instrumental for ensuring the acceptability of AI research and its successful integration into clinical practice in future.

## Appendix

Multimedia Appendix 1 Tables demonstrating the results of the multivariate regression analyses for the survey questions.

## Abbreviations

AI: artificial intelligence
A-Level: Advanced Level of Secondary Education
BAME: Black, Asian, and minority ethnic
GDPR: General Data Protection Regulations
HIPAA: Privacy Rules of the Health Insurance Portability and Accountability Act
ICO: Information Commissioner’s Office
ML: machine learning
NHS: National Health Service
