# Supplementary material for: Patient Perceptions on Data Sharing and Applying Artificial Intelligence to Health Care Data: Cross-sectional Survey
Source: J Med Internet Res. 2021 Aug 26;23(8):e26162. doi: 10.2196/26162 (PMC8430862; doi:10.2196/26162)
Supplement: Multimedia Appendix 1 [file jmir_v23i8e26162_app1.docx]

| Q1a | How much would you say you know about how the NHS uses health data for research purposes? | | | | | | |
| --- | --- | --- | --- | --- | --- | --- | --- |
|  |  | Coeff (B) | SE | Exp(B) | *p* value | 95% Confidence Interval | |
| Gender | Male | 0.237 | 0.1959 | 1.267 | 0.227 | 0.863 | 1.860 |
|  | Female | Reference | | | | | |
| Age | 16-30 | -0.277 | 0.3263 | 0.758 | 0.396 | 0.400 | 1.437 |
|  | 31-45 | -0.358 | 0.3291 | 0.699 | 0.277 | 0.367 | 1.332 |
|  | 46-64 | -0.640 | 0.2821 | 0.527 | 0.023 | 0.303 | 0.916 |
|  | 65+ | Reference | | | | | |
| Ethnicity | White | 0.410 | 0.2047 | 1.506 | 0.045 | 1.009 | 2.250 |
|  | BAME | Reference | | | | | |
| Education Levels | Low | -0.466 | 0.2281 | 0.627 | 0.041 | 0.401 | 0.981 |
|  | Medium | -0.028 | 0.2631 | 0.972 | 0.914 | 0.580 | 1.628 |
|  | High | Reference | | | | | |
| Health Status | Good | -0.108 | 0.1943 | 0.898 | 0.579 | 0.613 | 1.314 |
|  | Poor | Reference | | | | | |
| Internet Use | Daily | -0.028 | 0.3132 | 0.972 | 0.929 | 0.526 | 1.797 |
|  | < daily | Reference | | | | | |
| Smartphone Owner | Yes | 0.468 | 0.3135 | 1.597 | 0.136 | 0.864 | 2.952 |
|  | No | Reference | | | | | |

Multimedia Appendix 1: Tables demonstrating the results of the multivariate regression analyses for survey questions.

| Q1b | How much would you say you know about how commercial organisations use health data for research purposes? | | | | | | |
| --- | --- | --- | --- | --- | --- | --- | --- |
|  |  | Coeff (B) | SE | Exp(B) | *p* value | 95% Confidence Interval | |
| Gender | Male | 0.484 | 0.1983 | 1.623 | 0.015 | 1.100 | 2.393 |
|  | Female | Reference | | | | | |
| Age | 16-30 | -0.401 | 0.3268 | 0.669 | 0.219 | 0.353 | 1.270 |
|  | 31-45 | -0.299 | 0.3303 | 0.741 | 0.365 | 0.388 | 1.416 |
|  | 46-64 | -0.633 | 0.2836 | 0.531 | 0.026 | 0.305 | 0.926 |
|  | 65+ | Reference | | | | | |
| Ethnicity | White | 0.731 | 0.2092 | 2.077 | 0.001 | 1.379 | 3.130 |
|  | BAME | Reference | | | | | |
| Education Levels | Low | -0.720 | 0.2322 | 0.487 | 0.002 | 0.309 | 0.767 |
|  | Medium | -0.650 | 0.2658 | 0.522 | 0.015 | 0.310 | 0.879 |
|  | High | Reference | | | | | |
| Health Status | Good | -0.212 | 0.1962 | 0.809 | 0.279 | 0.551 | 1.188 |
|  | Poor | Reference | | | | | |
| Internet Use | Daily | -0.130 | 0.3163 | 0.878 | 0.680 | 0.472 | 1.631 |
|  | < daily | Reference | | | | | |
| Smartphone Owner | Yes | 0.663 | 0.3196 | 1.940 | 0.038 | 1.037 | 3.629 |
|  | No | Reference | | | | | |

| Q1c | How much would you say you know about how university researchers use health data for research purposes? | | | | | | |
| --- | --- | --- | --- | --- | --- | --- | --- |
|  |  | Coeff (B) | SE | Exp(B) | *p* value | 95% Confidence Interval | |
| Gender | Male | 0.346 | 0.1975 | 1.413 | 0.080 | 0.960 | 2.081 |
|  | Female | Reference | | | | | |
| Age | 16-30 | -0.749 | 0.3286 | 0.473 | 0.023 | 0.248 | 0.900 |
|  | 31-45 | -0.324 | 0.3310 | 0.723 | 0.328 | 0.378 | 1.384 |
|  | 46-64 | -0.596 | 0.2833 | 0.551 | 0.036 | 0.316 | 0.960 |
|  | 65+ | Reference | | | | | |
| Ethnicity | White | 0.603 | 0.2077 | 1.827 | 0.004 | 1.216 | 2.745 |
|  | BAME | Reference | | | | | |
| Education Levels | Low | -0.831 | 0.2328 | 0.436 | 0.001 | 0.276 | 0.688 |
|  | Medium | -0.473 | 0.2643 | 0.623 | 0.074 | 0.371 | 1.046 |
|  | High | Reference | | | | | |
| Health Status | Good | -0.187 | 0.1958 | 0.829 | 0.339 | 0.565 | 1.217 |
|  | Poor | Reference | | | | | |
| Internet Use | Daily | 0.167 | 0.3153 | 1.182 | 0.596 | 0.637 | 2.193 |
|  | < daily | Reference | | | | | |
| Smartphone Owner | Yes | 0.863 | 0.3184 | 2.369 | 0.007 | 1.269 | 4.422 |
|  | No | Reference | | | | | |

| Q2a | How likely would you be to allow your anonymised health information to be used for the purposes of medical research by the NHS? | | | | | | |
| --- | --- | --- | --- | --- | --- | --- | --- |
|  |  | Coeff (B) | SE | Exp(B) | *p* value | 95% Confidence Interval | |
| Gender | Male | 0.057 | 0.2094 | 1.059 | 0.784 | 0.703 | 1.597 |
|  | Female | Reference | | | | | |
| Age | 16-30 | -0.513 | 0.3584 | 0.599 | 0.152 | 0.297 | 1.209 |
|  | 31-45 | -0.898 | 0.3601 | 0.407 | 0.013 | 0.201 | 0.825 |
|  | 46-64 | -0.324 | 0.3098 | 0.723 | 0.296 | 0.394 | 1.328 |
|  | 65+ | Reference | | | | | |
| Ethnicity | White | 1.138 | 0.2199 | 3.120 | 0.001 | 2.207 | 4.800 |
|  | BAME | Reference | | | | | |
| Education Levels | Low | -0.576 | 0.2446 | 0.562 | 0.019 | 0.348 | 0.908 |
|  | Medium | -0.246 | 0.2826 | 0.782 | 0.385 | 0.449 | 1.361 |
|  | High | Reference | | | | | |
| Health Status | Good | 0.172 | 0.2089 | 1.188 | 0.977 | 0.517 | 1.899 |
|  | Poor | Reference | | | | | |
| Internet Use | Daily | -0.010 | 0.3321 | 0.991 | 0.977 | 0.517 | 1.899 |
|  | < daily | Reference | | | | | |
| Smartphone Owner | Yes | 0.814 | 0.3329 | 2.258 | 0.014 | 1.176 | 4.336 |
|  | No | Reference | | | | | |

| Q2b | How likely would you be to allow your anonymised health information to be used for the purposes of medical research by commercial organisations? | | | | | | |
| --- | --- | --- | --- | --- | --- | --- | --- |
|  |  | Coeff (B) | SE | Exp(B) | *p* value | 95% Confidence Interval | |
| Gender | Male | 0.299 | 0.1968 | 1.349 | 0.128 | 0.917 | 1.983 |
|  | Female | Reference | | | | | |
| Age | 16-30 | 0.666 | 0.3270 | 1.946 | 0.042 | 1.025 | 3.693 |
|  | 31-45 | 0.253 | 0.3297 | 1.288 | 0.443 | 0.675 | 2.458 |
|  | 46-64 | 0.438 | 0.2812 | 1.550 | 0.119 | 0.893 | 2.690 |
|  | 65+ | Reference | | | | | |
| Ethnicity | White | 0.272 | 0.2054 | 1.313 | 0.185 | 0.878 | 1.963 |
|  | BAME | Reference | | | | | |
| Education Levels | Low | -0.392 | 0.2294 | 0.675 | 0.087 | 0.431 | 1.059 |
|  | Medium | -0.103 | 0.2627 | 0.902 | 0.694 | 0.539 | 1.509 |
|  | High | Reference | | | | | |
| Health Status | Good | 0.214 | 0.1952 | 1.238 | 0.274 | 0.844 | 1.815 |
|  | Poor | Reference | | | | | |
| Internet Use | Daily | -0.387 | 0.3410 | 0.679 | 0.218 | 0.367 | 1.257 |
|  | < daily | Reference | | | | | |
| Smartphone Owner | Yes | 0.032 | 0.3133 | 1.032 | 0.920 | 0.558 | 1.907 |
|  | No | Reference | | | | | |

| Q2c | How likely would you be to allow your anonymised health information to be used for the purposes of medical research by university researchers? | | | | | | |
| --- | --- | --- | --- | --- | --- | --- | --- |
|  |  | Coeff (B) | SE | Exp(B) | *p* value | 95% Confidence Interval | |
| Gender | Male | -0.124 | 0.2014 | 0.883 | 0.538 | 0.595 | 1.311 |
|  | Female | Reference | | | | | |
| Age | 16-30 | -0.026 | 0.3354 | 0.974 | 0.937 | 0.505 | 1.879 |
|  | 31-45 | -0.481 | 0.3385 | 0.618 | 0.155 | 0.318 | 1.200 |
|  | 46-64 | 0.083 | 0.2887 | 1.087 | 0.774 | 0.617 | 1.913 |
|  | 65+ | Reference | | | | | |
| Ethnicity | White | 1.015 | 0.2137 | 2.759 | 0.001 | 1.815 | 4.195 |
|  | BAME | Reference | | | | | |
| Education Levels | Low | -0.710 | 0.2364 | 0.491 | 0.003 | 0.309 | 0.781 |
|  | Medium | -0.461 | 0.2696 | 0.630 | 0.087 | 0.372 | 1.069 |
|  | High | Reference | | | | | |
| Health Status | Good | 0.051 | 0.2001 | 1.052 | 0.800 | 0.711 | 1.557 |
|  | Poor | Reference | | | | | |
| Internet Use | Daily | -0.379 | 0.3218 | 0.685 | 0.239 | 0.364 | 1.287 |
|  | < daily | Reference | | | | | |
| Smartphone Owner | Yes | 0.860 | 0.3198 | 2.363 | 0.007 | 1.263 | 4.423 |
|  | No | Reference | | | | | |

| Q4a | Which of the following types of health data would you be comfortable sharing with university researchers? Radiology images | | | | | | |
| --- | --- | --- | --- | --- | --- | --- | --- |
|  |  | Coeff (B) | SE | Exp(B) | *p* value | 95% Confidence Interval | |
| Gender | Male | -0.018 | 0.3195 | 0.983 | 0.956 | 0.525 | 1.838 |
|  | Female | Reference | | | | | |
| Age | 16-30 | -1.113 | 0.5094 | 0.329 | 0.029 | 0.121 | 0.892 |
|  | 31-45 | 0.741 | 0.6469 | 2.097 | 0.252 | 0.590 | 7.452 |
|  | 46-64 | 0.197 | 0.4764 | 1.218 | 0.679 | 0.479 | 3.098 |
|  | 65+ | Reference | | | | | |
| Ethnicity | White | 1.000 | 0.3307 | 2.719 | 0.002 | 1.422 | 5.199 |
|  | BAME | Reference | | | | | |
| Education Levels | Low | 0.752 | 0.4125 | 2.122 | 0.068 | 0.945 | 4.764 |
|  | Medium | -0.087 | 0.3939 | 0.917 | 0.825 | 0.424 | 1.984 |
|  | High | Reference | | | | | |
| Health Status | Good | -0.133 | 0.3169 | 0.875 | 0.674 | 0.470 | 1.629 |
|  | Poor | Reference | | | | | |
| Internet Use | Daily | 0.030 | 0.5242 | 1.031 | 0.954 | 0.369 | 2.880 |
|  | < daily | Reference | | | | | |
| Smartphone Owner | Yes | 1.847 | 0.4917 | 6.339 | 0.001 | 2.418 | 16.615 |
|  | No | Reference | | | | | |

| Q4b | Which of the following types of health data would you be comfortable sharing with university researchers? Blood test results | | | | | | |
| --- | --- | --- | --- | --- | --- | --- | --- |
|  |  | Coeff (B) | SE | Exp(B) | *p* value | 95% Confidence Interval | |
| Gender | Male | -0.193 | 0.2929 | 0.824 | 0.510 | 0.464 | 1.464 |
|  | Female | Reference | | | | | |
| Age | 16-30 | -0.238 | 0.4742 | 0.788 | 0.616 | 0.311 | 1.997 |
|  | 31-45 | 0.279 | 0.5036 | 1.322 | 0.580 | 0.493 | 3.547 |
|  | 46-64 | 0.429 | 0.4227 | 1.535 | 0.311 | 0.670 | 3.515 |
|  | 65+ | Reference | | | | | |
| Ethnicity | White | 1.369 | 0.3106 | 3.929 | 0.001 | 2.138 | 7.223 |
|  | BAME | Reference | | | | | |
| Education Levels | Low | -0.116 | 0.3458 | 0.890 | 0.737 | 0.452 | 1.753 |
|  | Medium | -0.029 | 0.3891 | 0.972 | 0.941 | 0.453 | 2.083 |
|  | High | Reference | | | | | |
| Health Status | Good | -0.058 | 0.2908 | 0.944 | 0.843 | 0.534 | 1.669 |
|  | Poor | Reference | | | | | |
| Internet Use | Daily | 0.281 | 0.4347 | 1.324 | 0.519 | 0.565 | 3.104 |
|  | < daily | Reference | | | | | |
| Smartphone Owner | Yes | 1.139 | 0.4337 | 3.124 | 0.009 | 1.335 | 7.308 |
|  | No | Reference | | | | | |

| Q4c | Which of the following types of health data would you be comfortable sharing with university researchers? Free text clinical notes | | | | | | |
| --- | --- | --- | --- | --- | --- | --- | --- |
|  |  | Coeff (B) | SE | Exp(B) | *p* value | 95% Confidence Interval | |
| Gender | Male | 0.138 | 0.2269 | 1.148 | 0.543 | 0.736 | 1.791 |
|  | Female | Reference | | | | | |
| Age | 16-30 | -0.958 | 0.3808 | 0.384 | 0.012 | 0.182 | 0.809 |
|  | 31-45 | -0.333 | 0.3823 | 0.717 | 0.384 | 0.339 | 1.517 |
|  | 46-64 | -0.182 | 0.3271 | 0.833 | 0.577 | 0.439 | 1.582 |
|  | 65+ | Reference | | | | | |
| Ethnicity | White | 0.746 | 0.2356 | 2.110 | 0.002 | 1.329 | 3.347 |
|  | BAME | Reference | | | | | |
| Education Levels | Low | -0.229 | 0.2642 | 0.795 | 0.385 | 0.474 | 1.334 |
|  | Medium | -0.470 | 0.3075 | 0.625 | 0.127 | 0.342 | 1.142 |
|  | High | Reference | | | | | |
| Health Status | Good | 0.184 | 0.2265 | 1.202 | 0.416 | 0.771 | 1.874 |
|  | Poor | Reference | | | | | |
| Internet Use | Daily | 0.133 | 0.3600 | 1.142 | 0.712 | 0.564 | 2.313 |
|  | < daily | Reference | | | | | |
| Smartphone Owner | Yes | 0.587 | 0.3609 | 1.799 | 0.104 | 0.887 | 3.649 |
|  | No | Reference | | | | | |

| Q4d | Which of the following types of health data would you be comfortable sharing with university researchers? Diagnoses | | | | | | |
| --- | --- | --- | --- | --- | --- | --- | --- |
|  |  | Coeff (B) | SE | Exp(B) | *p* value | 95% Confidence Interval | |
| Gender | Male | 0.102 | 0.2654 | 1.108 | 0.700 | 0.658 | 1.863 |
|  | Female | Reference | | | | | |
| Age | 16-30 | -0.399 | 0.4487 | 0.671 | 0.374 | 0.278 | 1.617 |
|  | 31-45 | -0.058 | 0.4712 | 0.944 | 0.902 | 0.375 | 2.377 |
|  | 46-64 | -0.259 | 0.3962 | 0.772 | 0.514 | 0.355 | 1.679 |
|  | 65+ | Reference | | | | | |
| Ethnicity | White | 1.053 | 0.2723 | 2.867 | 0.001 | 1.682 | 4.890 |
|  | BAME | Reference | | | | | |
| Education Levels | Low | -0.284 | 0.3138 | 0.753 | 0.365 | 0.407 | 1.392 |
|  | Medium | -0.448 | 0.3456 | 0.639 | 0.195 | 0.325 | 1.258 |
|  | High | Reference | | | | | |
| Health Status | Good | 0.345 | 0.2685 | 1.411 | 0.200 | 0.834 | 2.389 |
|  | Poor | Reference | | | | | |
| Internet Use | Daily | -0.1787 | 0.4303 | 0.837 | 0.679 | 0.360 | 1.945 |
|  | < daily | Reference | | | | | |
| Smartphone Owner | Yes | 0.549 | 0.41844 | 1.732 | 0.189 | 0.763 | 3.933 |
|  | No | Reference | | | | | |

| Q4e | Which of the following types of health data would you be comfortable sharing with university researchers? Vital signs | | | | | | |
| --- | --- | --- | --- | --- | --- | --- | --- |
|  |  | Coeff (B) | SE | Exp(B) | *p* value | 95% Confidence Interval | |
| Gender | Male | 0.022 | 0.2454 | 1.023 | 0.927 | 0.632 | 1.654 |
|  | Female | Reference | | | | | |
| Age | 16-30 | -0.737 | 0.4060 | 0.478 | 0.069 | 0.216 | 1.060 |
|  | 31-45 | 0.029 | 0.4270 | 1.029 | 0.947 | 0.446 | 2.376 |
|  | 46-64 | -0.038 | 0.3601 | 0.963 | 0.916 | 0.475 | 1.950 |
|  | 65+ | Reference | | | | | |
| Ethnicity | White | 1.175 | 0.2516 | 3.238 | 0.001 | 1.978 | 5.302 |
|  | BAME | Reference | | | | | |
| Education Levels | Low | -0.255 | 0.2876 | 0.775 | 0.375 | 0.441 | 1.362 |
|  | Medium | -0.190 | 0.3272 | 0.827 | 0.561 | 0.435 | 1.570 |
|  | High | Reference | | | | | |
| Health Status | Good | 0.209 | 0.2465 | 1.232 | 0.397 | 0.760 | 1.997 |
|  | Poor | Reference | | | | | |
| Internet Use | Daily | 0.258 | 0.3806 | 1.295 | 0.499 | 0.614 | 2.718 |
|  | < daily | Reference | | | | | |
| Smartphone Owner | Yes | 0.684 | 0.3839 | 1.982 | 0.075 | 0.934 | 4.207 |
|  | No | Reference | | | | | |

| Q4f | Which of the following types of health data would you be comfortable sharing with university researchers? Clinic letters | | | | | | |
| --- | --- | --- | --- | --- | --- | --- | --- |
|  |  | Coeff (B) | SE | Exp(B) | *p* value | 95% Confidence Interval | |
| Gender | Male | 0.043 | 0.2258 | 1.044 | 0.850 | 0.670 | 1.624 |
|  | Female | Reference | | | | | |
| Age | 16-30 | -0.974 | 0.3771 | 0.378 | 0.010 | 0.180 | 0.790 |
|  | 31-45 | -0.473 | 0.3767 | 0.623 | 0.209 | 0.298 | 1.303 |
|  | 46-64 | -0.028 | 0.3234 | 0.972 | 0.931 | 0.516 | 1.833 |
|  | 65+ | Reference | | | | | |
| Ethnicity | White | 0.604 | 0.2337 | 1.829 | 0.010 | 1.157 | 2.892 |
|  | BAME | Reference | | | | | |
| Education Levels | Low | -0.092 | 0.2625 | 0.912 | 0.725 | 0.545 | 1.526 |
|  | Medium | -0.280 | 0.3054 | 0.755 | 0.359 | 0.415 | 1.375 |
|  | High | Reference | | | | | |
| Health Status | Good | 0.051 | 0.2246 | 1.052 | 0.832 | 0.677 | 1.634 |
|  | Poor | Reference | | | | | |
| Internet Use | Daily | 0.212 | 0.3575 | 1.236 | 0.554 | 0.613 | 2.490 |
|  | < daily | Reference | | | | | |
| Smartphone Owner | Yes | 0.412 | 0.3577 | 1.510 | 0.249 | 0.749 | 3.044 |
|  | No | Reference | | | | | |

| Q4g | Which of the following types of health data would you be comfortable sharing with university researchers? Operations and treatments | | | | | | |
| --- | --- | --- | --- | --- | --- | --- | --- |
|  |  | Coeff (B) | SE | Exp(B) | *p* value | 95% Confidence Interval | |
| Gender | Male | 0.051 | 0.2591 | 1.053 | 0.843 | 0.634 | 1.749 |
|  | Female | Reference | | | | | |
| Age | 16-30 | -0.994 | 0.4461 | 0.370 | 0.026 | 0.154 | 0.887 |
|  | 31-45 | -0.348 | 0.4710 | 0.706 | 0.460 | 0.281 | 1.778 |
|  | 46-64 | -0.362 | 0.3974 | 0.696 | 0.362 | 0.319 | 1.517 |
|  | 65+ | Reference | | | | | |
| Ethnicity | White | 0.800 | 0.2641 | 2.226 | 0.002 | 1.326 | 3.736 |
|  | BAME | Reference | | | | | |
| Education Levels | Low | -0.368 | 0.3063 | 0.692 | 0.230 | 0.380 | 1.262 |
|  | Medium | -0.506 | 0.3384 | 0.603 | 0.135 | 0.311 | 1.170 |
|  | High | Reference | | | | | |
| Health Status | Good | 0.088 | 0.2592 | 1.093 | 0.733 | 0.657 | 1.816 |
|  | Poor | Reference | | | | | |
| Internet Use | Daily | -0.046 | 0.4167 | 0.955 | 0.913 | 0.422 | 2.162 |
|  | < daily | Reference | | | | | |
| Smartphone Owner | Yes | 0.776 | 0.4079 | 2.173 | 0.057 | 0.97 | 4.833 |
|  | No | Reference | | | | | |

| Q4h | Which of the following types of health data would you be comfortable sharing with university researchers? Medications | | | | | | |
| --- | --- | --- | --- | --- | --- | --- | --- |
|  |  | Coeff (B) | SE | Exp(B) | *p* value | 95% Confidence Interval | |
| Gender | Male | 0.105 | 0.2773 | 1.111 | 0.705 | 0.645 | 1.913 |
|  | Female | Reference | | | | | |
| Age | 16-30 | -0.502 | 0.4851 | 0.605 | 0.301 | 0.234 | 1.566 |
|  | 31-45 | -0.586 | 0.4930 | 0.556 | 0.234 | 0.212 | 1.462 |
|  | 46-64 | -0.322 | 0.4161 | 0.725 | 0.439 | 0.321 | 1.639 |
|  | 65+ | Reference | | | | | |
| Ethnicity | White | 0.988 | 0.2879 | 2.686 | 0.001 | 1.528 | 4.722 |
|  | BAME | Reference | | | | | |
| Education Levels | Low | -0.857 | 0.3319 | 0.424 | 0.010 | 0.221 | 0.813 |
|  | Medium | -0.790 | 0.3709 | 0.454 | 0.033 | 0.219 | 0.939 |
|  | High | Reference | | | | | |
| Health Status | Good | 0.403 | 0.2816 | 1.497 | 0.152 | 0.862 | 2.599 |
|  | Poor | Reference | | | | | |
| Internet Use | Daily | 0.011 | 0.4210 | 1.011 | 0.979 | 0.443 | 2.308 |
|  | < daily | Reference | | | | | |
| Smartphone Owner | Yes | 0.987 | 0.4175 | 2.683 | 0.018 | 1.184 | 6.082 |
|  | No | Reference | | | | | |

| Q5 | To what extent would you support the university creating an anonymised dataset of routinely collected healthcare data for research purposes? | | | | | | |
| --- | --- | --- | --- | --- | --- | --- | --- |
|  |  | Coeff (B) | SE | Exp(B) | *p* value | 95% Confidence Interval | |
| Gender | Male | 0.052 | 0.2111 | 1.053 | 0.805 | 0.696 | 1.594 |
|  | Female | Reference | | | | | |
| Age | 16-30 | -1.116 | 0.3580 | 0.328 | 0.002 | 0.162 | 0.661 |
|  | 31-45 | -1.096 | 0.3609 | 0.334 | 0.002 | 0.165 | 0.678 |
|  | 46-64 | -0.257 | 0.3068 | 0.773 | 0.402 | 0.424 | 1.411 |
|  | 65+ | Reference | | | | | |
| Ethnicity | White | 0.612 | 0.2214 | 1.844 | 0.006 | 1.195 | 2.846 |
|  | BAME | Reference | | | | | |
| Education Levels | Low | -0.725 | 0.2480 | 0.484 | 0.003 | 0.298 | 0.788 |
|  | Medium | -0.437 | 0.2845 | 0.646 | 0.124 | 0.370 | 1.128 |
|  | High | Reference | | | | | |
| Health Status | Good | -0.062 | 0.2098 | 0.940 | 0.767 | 0.623 | 1.418 |
|  | Poor | Reference | | | | | |
| Internet Use | Daily | -0.288 | 0.3394 | 0.750 | 0.396 | 0.386 | 1.458 |
|  | < daily | Reference | | | | | |
| Smartphone Owner | Yes | 0.721 | 0.3390 | 2.057 | 0.033 | 1.059 | 3.999 |
|  | No | Reference | | | | | |

| Q6 | To what extent would you support the transfer of health data to a university if there was a very small chance of it being re-identified? | | | | | | |
| --- | --- | --- | --- | --- | --- | --- | --- |
|  |  | Coeff (B) | SE | Exp(B) | *p* value | 95% Confidence Interval | |
| Gender | Male | -0.009 | 0.1968 | 0.991 | 0.965 | 0.674 | 1.458 |
|  | Female | Reference | | | | | |
| Age | 16-30 | -0.508 | 0.3305 | 0.602 | 0.124 | 0.315 | 1.150 |
|  | 31-45 | -0.882 | 0.3335 | 0.414 | 0.008 | 0.215 | 0.796 |
|  | 46-64 | -0.348 | 0.2836 | 0.706 | 0.220 | 0.405 | 1.231 |
|  | 65+ | Reference | | | | | |
| Ethnicity | White | 0.326 | 0.2055 | 1.386 | 0.112 | 0.926 | 2.073 |
|  | BAME | Reference | | | | | |
| Education Levels | Low | -0.407 | 0.2304 | 0.666 | 0.077 | 0.424 | 1.045 |
|  | Medium | -0.392 | 0.2646 | 0.676 | 0.138 | 0.402 | 1.135 |
|  | High | Reference | | | | | |
| Health Status | Good | 0.124 | 0.1957 | 1.132 | 0.525 | 0.772 | 1.662 |
|  | Poor | Reference | | | | | |
| Internet Use | Daily | -0.008 | 0.3148 | 0.992 | 0.979 | 0.535 | 1.838 |
|  | < daily | Reference | | | | | |
| Smartphone Owner | Yes | 0.479 | 0.3153 | 1.614 | 0.129 | 0.870 | 2.994 |
|  | No | Reference | | | | | |

| Q8 | To what extent do you agree with the fact that researchers are legally allowed to access anonymised health data without patient consent? | | | | | | |
| --- | --- | --- | --- | --- | --- | --- | --- |
|  |  | Coeff (B) | SE | Exp(B) | *p* value | 95% Confidence Interval | |
| Gender | Male | 0.068 | 0.1985 | 1.071 | 0.731 | 0.726 | 1.580 |
|  | Female | Reference | | | | | |
| Age | 16-30 | -1.553 | 0.3406 | 0.212 | 0.001 | 0.109 | 0.412 |
|  | 31-45 | -1.193 | 0.3407 | 0.303 | 0.001 | 0.156 | 0.591 |
|  | 46-64 | -0.773 | 0.2897 | 0.461 | 0.008 | 0.262 | 0.814 |
|  | 65+ | Reference | | | | | |
| Ethnicity | White | 0.693 | 0.2088 | 2.000 | 0.001 | 1.328 | 3.011 |
|  | BAME | Reference | | | | | |
| Education Levels | Low | -0.244 | 0.2309 | 0.783 | 0.290 | 0.498 | 1.231 |
|  | Medium | 0.112 | 0.2668 | 1.119 | 0.675 | 0.663 | 1.887 |
|  | High | Reference | | | | | |
| Health Status | Good | 0.009 | 0.1971 | 1.573 | 0.154 | 0.855 | 2.931 |
|  | Poor | Reference | | | | | |
| Internet Use | Daily | 0.453 | 0.3175 | 1.573 | 0.154 | 0.844 | 2.931 |
|  | < daily | Reference | | | | | |
| Smartphone Owner | Yes | 0.846 | 0.3201 | 2.331 | 0.008 | 1.245 | 4.366 |
|  | No | Reference | | | | | |

| Q9a | Which of the following types of organisations would you be happy to allow access to the data for research purposes? Drug/pharmaceutical company | | | | | | |
| --- | --- | --- | --- | --- | --- | --- | --- |
|  |  | Coeff (B) | SE | Exp(B) | *p* value | 95% Confidence Interval | |
| Gender | Male | 0.044 | 0.2309 | 1.044 | 0.851 | 0.664 | 1.642 |
|  | Female | Reference | | | | | |
| Age | 16-30 | 0.387 | 0.3910 | 1.473 | 0.322 | 0.684 | 3.168 |
|  | 31-45 | -0.565 | 0.3877 | 0.568 | 0.145 | 0.266 | 1.215 |
|  | 46-64 | -0.598 | 0.3382 | 0.550 | 0.077 | 0.283 | 1.067 |
|  | 65+ | Reference | | | | | |
| Ethnicity | White | 0.837 | 0.2469 | 2.309 | 0.001 | 1.423 | 3.747 |
|  | BAME | Reference | | | | | |
| Education Levels | Low | -0.488 | 0.2697 | 0.614 | 0.071 | 0.362 | 1.042 |
|  | Medium | -0.202 | 0.3071 | 0.817 | 0.510 | 0.447 | 1.491 |
|  | High | Reference | | | | | |
| Health Status | Good | 0.061 | 0.2297 | 1.063 | 0.789 | 0.678 | 1.668 |
|  | Poor | Reference | | | | | |
| Internet Use | Daily | 0.387 | 0.3861 | 1.472 | 0.316 | 0.691 | 3.138 |
|  | < daily | Reference | | | | | |
| Smartphone Owner | Yes | 0.994 | 0.3939 | 2.702 | 0.012 | 1.248 | 5.847 |
|  | No | Reference | | | | | |

| Q9b | Which of the following types of organisations would you be happy to allow access to the data for research purposes? News organisation | | | | | | |
| --- | --- | --- | --- | --- | --- | --- | --- |
|  |  | Coeff (B) | SE | Exp(B) | *p* value | 95% Confidence Interval | |
| Gender | Male | -0.141 | 0.4330 | 0.869 | 0.745 | 0.372 | 2.030 |
|  | Female | Reference | | | | | |
| Age | 16-30 | -0.064 | 0.5931 | 0.938 | 0.915 | 0.293 | 3.001 |
|  | 31-45 | -1.513 | 0.7567 | 0.220 | 0.046 | 0.050 | 0.971 |
|  | 46-64 | -1.543 | 0.6376 | 0.214 | 0.016 | 0.061 | 0.746 |
|  | 65+ | Reference | | | | | |
| Ethnicity | White | 0.197 | 0.4488 | 1.218 | 0.660 | 0.505 | 2.936 |
|  | BAME | Reference | | | | | |
| Education Levels | Low | -0.342 | 0.4804 | 0.710 | 0.477 | 0.277 | 1.821 |
|  | Medium | -2.202 | 1.0525 | 0.111 | 0.036 | 0.014 | 0.870 |
|  | High | Reference | | | | | |
| Health Status | Good | 0.181 | 0.4165 | 1.198 | 0.664 | 0.530 | 2.710 |
|  | Poor | Reference | | | | | |
| Internet Use | Daily | -0.169 | 0.6962 | 0.845 | 0.808 | 0.216 | 3.306 |
|  | < daily | Reference | | | | | |
| Smartphone Owner | Yes | -0.001 | 0.6404 | 0.999 | 0.998 | 0.285 | 3.504 |
|  | No | Reference | | | | | |

| Q9c | Which of the following types of organisations would you be happy to allow access to the data for research purposes? Other hospital | | | | | | |
| --- | --- | --- | --- | --- | --- | --- | --- |
|  |  | Coeff (B) | SE | Exp(B) | *p* value | 95% Confidence Interval | |
| Gender | Male | 0.222 | 0.2504 | 1.249 | 0.375 | 0.765 | 2.040 |
|  | Female | Reference | | | | | |
| Age | 16-30 | -0.583 | 0.4125 | 0.558 | 0.158 | 0.249 | 1.253 |
|  | 31-45 | -0.136 | 0.4256 | 0.873 | 0.749 | 0.379 | 2.010 |
|  | 46-64 | 0.247 | 0.3699 | 1.280 | 0.505 | 0.620 | 2.642 |
|  | 65+ | Reference | | | | | |
| Ethnicity | White | 1.053 | 0.2557 | 2.866 | 0.001 | 1.736 | 4.732 |
|  | BAME | Reference | | | | | |
| Education Levels | Low | -0.029 | 0.2945 | 0.971 | 0.921 | 0.545 | 1.729 |
|  | Medium | -0.011 | 0.3336 | 0.989 | 0.973 | 0.514 | 1.902 |
|  | High | Reference | | | | | |
| Health Status | Good | -0.042 | 0.2482 | 0.959 | 0.866 | 0.590 | 1.560 |
|  | Poor | Reference | | | | | |
| Internet Use | Daily | 0.489 | 0.3807 | 1.631 | 0.199 | 0.773 | 3.439 |
|  | < daily | Reference | | | | | |
| Smartphone Owner | Yes | 1.015 | 0.3876 | 2.760 | 0.009 | 1.291 | 5.899 |
|  | No | Reference | | | | | |

| Q9d | Which of the following types of organisations would you be happy to allow access to the data for research purposes? Medical technology manufacturer | | | | | | |
| --- | --- | --- | --- | --- | --- | --- | --- |
|  |  | Coeff (B) | SE | Exp(B) | *p* value | 95% Confidence Interval | |
| Gender | Male | 0.179 | 0.2251 | 1.196 | 0.427 | 0.769 | 1.859 |
|  | Female | Reference | | | | | |
| Age | 16-30 | 0.586 | 0.3753 | 1.796 | 0.119 | 0.861 | 3.748 |
|  | 31-45 | -0.010 | 0.3757 | 0.990 | 0.978 | 0.474 | 2.067 |
|  | 46-64 | 0.029 | 0.3230 | 1.030 | 0.927 | 0.547 | 1.940 |
|  | 65+ | Reference | | | | | |
| Ethnicity | White | 0.458 | 0.2361 | 1.581 | 0.052 | 0.995 | 2.511 |
|  | BAME | Reference | | | | | |
| Education Levels | Low | -0.492 | 0.2606 | 0.611 | 0.059 | 0.367 | 1.018 |
|  | Medium | -0.373 | 0.3002 | 0.689 | 0.214 | 0.382 | 1.241 |
|  | High | Reference | | | | | |
| Health Status | Good | -0.090 | 0.2229 | 0.914 | 0.686 | 0.590 | 1.414 |
|  | Poor | Reference | | | | | |
| Internet Use | Daily | 0.099 | 0.3651 | 1.104 | 0.786 | 0.540 | 2.258 |
|  | < daily | Reference | | | | | |
| Smartphone Owner | Yes | 0.476 | 0.3685 | 1.609 | 0.197 | 0.781 | 3.313 |
|  | No | Reference | | | | | |

| Q9e | Which of the following types of organisations would you be happy to allow access to the data for research purposes? Technology company | | | | | | |
| --- | --- | --- | --- | --- | --- | --- | --- |
|  |  | Coeff (B) | SE | Exp(B) | *p* value | 95% Confidence Interval | |
| Gender | Male | -0.003 | 0.2642 | 0.997 | 0.990 | 0.594 | 1.673 |
|  | Female | Reference | | | | | |
| Age | 16-30 | 0.170 | 0.4125 | 1.186 | 0.680 | 0.528 | 2.662 |
|  | 31-45 | -0.697 | 0.4520 | 0.498 | 0.123 | 0.205 | 1.208 |
|  | 46-64 | -0.395 | 0.3705 | 0.674 | 0.286 | 0.326 | 1.392 |
|  | 65+ | Reference | | | | | |
| Ethnicity | White | 0.276 | 0.2799 | 1.318 | 0.324 | 0.761 | 2.281 |
|  | BAME | Reference | | | | | |
| Education Levels | Low | -0.508 | 0.3139 | 0.601 | 0.105 | 0.325 | 1.113 |
|  | Medium | -0.321 | 0.3519 | 0.716 | 0.362 | 0.364 | 1.446 |
|  | High | Reference | | | | | |
| Health Status | Good | 0.148 | 0.2615 | 1.159 | 0.572 | 0.694 | 1.936 |
|  | Poor | Reference | | | | | |
| Internet Use | Daily | -0.132 | 0.4269 | 0.877 | 0.758 | 0.380 | 2.024 |
|  | < daily | Reference | | | | | |
| Smartphone Owner | Yes | -0.018 | 0.4213 | 0.983 | 0.967 | 0.430 | 2.244 |
|  | No | Reference | | | | | |

| Q9f | Which of the following types of organisations would you be happy to allow access to the data for research purposes? Insurance Company | | | | | | |
| --- | --- | --- | --- | --- | --- | --- | --- |
|  |  | Coeff (B) | SE | Exp(B) | *p* value | 95% Confidence Interval | |
| Gender | Male | 0.777 | 0.4475 | 2.175 | 0.083 | 0.905 | 5.228 |
|  | Female | Reference | | | | | |
| Age | 16-30 | -0.110 | 0.6563 | 0.895 | 0.866 | 0.247 | 3.241 |
|  | 31-45 | -0.782 | 0.7689 | 0.457 | 0.309 | 0.101 | 2.065 |
|  | 46-64 | -0.403 | 0.5507 | 0.668 | 0.464 | 0.227 | 1.967 |
|  | 65+ | Reference | | | | | |
| Ethnicity | White | 0.920 | 0.5357 | 2.510 | 0.086 | 0.878 | 7.172 |
|  | BAME | Reference | | | | | |
| Education Levels | Low | -0.344 | 0.5610 | 0.709 | 0.505 | 0.258 | 1.949 |
|  | Medium | -0.144 | 0.5750 | 0.892 | 0.843 | 0.289 | 2.754 |
|  | High | Reference | | | | | |
| Health Status | Good | 0.126 | 0.4315 | 1.134 | 0.770 | 0.487 | 2.643 |
|  | Poor | Reference | | | | | |
| Internet Use | Daily | 0.011 | 0.6780 | 1.011 | 0.987 | 0.268 | 3.820 |
|  | < daily | Reference | | | | | |
| Smartphone Owner | Yes | -0.468 | 0.6133 | 0.626 | 0.445 | 0.188 | 2.083 |
|  | No | Reference | | | | | |

| Q9g | Which of the following types of organisations would you be happy to allow access to the data for research purposes? Other university | | | | | | |
| --- | --- | --- | --- | --- | --- | --- | --- |
|  |  | Coeff (B) | SE | Exp(B) | *p* value | 95% Confidence Interval | |
| Gender | Male | -0.516 | 0.2356 | 0.597 | 0.029 | 0.376 | 0.947 |
|  | Female | Reference | | | | | |
| Age | 16-30 | -0.498 | 0.3882 | 0.608 | 0.200 | 0.284 | 1.301 |
|  | 31-45 | -0.134 | 0.3900 | 0.875 | 0.731 | 0.407 | 1.879 |
|  | 46-64 | -0.213 | 0.3333 | 0.808 | 0.522 | 0.420 | 1.552 |
|  | 65+ | Reference | | | | | |
| Ethnicity | White | 1.008 | 0.2476 | 2.741 | 0.001 | 1.687 | 4.453 |
|  | BAME | Reference | | | | | |
| Education Levels | Low | -0.763 | 0.2700 | 0.466 | 0.005 | 0.275 | 0.791 |
|  | Medium | -0.745 | 0.3163 | 0.475 | 0.019 | 0.255 | 0.883 |
|  | High | Reference | | | | | |
| Health Status | Good | -0.195 | 0.2328 | 0.823 | 0.403 | 0.522 | 1.299 |
|  | Poor | Reference | | | | | |
| Internet Use | Daily | -0.063 | 0.3758 | 0.939 | 0.866 | 0.449 | 1.961 |
|  | < daily | Reference | | | | | |
| Smartphone Owner | Yes | 0.838 | 0.3829 | 2.313 | 0.029 | 1.092 | 4.899 |
|  | No | Reference | | | | | |

| Q10 | How much would you say you know about ‘artificial intelligence’? | | | | | | |
| --- | --- | --- | --- | --- | --- | --- | --- |
|  |  | Coeff (B) | SE | Exp(B) | *p* value | 95% Confidence Interval | |
| Gender | Male | 0.365 | 0.1983 | 1.440 | 0.066 | 0.976 | 2.214 |
|  | Female | Reference | | | | | |
| Age | 16-30 | -0.081 | 0.3298 | 0.922 | 0.807 | 0.483 | 1.760 |
|  | 31-45 | -0.356 | 0.2649 | 0.729 | 0.232 | 0.433 | 1.224 |
|  | 46-64 | -0.154 | 0.2857 | 0.858 | 0.591 | 0.490 | 1.501 |
|  | 65+ | Reference | | | | | |
| Ethnicity | White | 0.614 | 0.2085 | 1.848 | 0.003 | 1.228 | 2.781 |
|  | BAME | Reference | | | | | |
| Education Levels | Low | -0.843 | 0.2335 | 0.431 | 0.001 | 0.272 | 0.680 |
|  | Medium | -0.317 | 0.2649 | 0.729 | 0.232 | 0.433 | 1.224 |
|  | High | Reference | | | | | |
| Health Status | Good | -0.102 | 0.1968 | 0.903 | 0.605 | 0.614 | 1.328 |
|  | Poor | Reference | | | | | |
| Internet Use | Daily | 0.578 | 0.3214 | 1.783 | 0.072 | 0.950 | 3.348 |
|  | < daily | Reference | | | | | |
| Smartphone Owner | Yes | 1.177 | 0.3259 | 3.244 | 0.001 | 1.713 | 6.144 |
|  | No | Reference | | | | | |

| Q11 | How much would you say you know about ‘machine learning? | | | | | | |
| --- | --- | --- | --- | --- | --- | --- | --- |
|  |  | Coeff (B) | SE | Exp(B) | *p* value | 95% Confidence Interval | |
| Gender | Male | 0.443 | 0.1981 | 1.557 | 0.025 | 1.056 | 2.297 |
|  | Female | Reference | | | | | |
| Age | 16-30 | -0.042 | 0.3282 | 0.959 | 0.989 | 0.504 | 1.825 |
|  | 31-45 | -0.363 | 0.3319 | 0.695 | 0.274 | 0.363 | 1.333 |
|  | 46-64 | -0.272 | 0.2856 | 0.762 | 0.341 | 0.435 | 1.333 |
|  | 65+ | Reference | | | | | |
| Ethnicity | White | 0.455 | 0.2073 | 1.576 | 0.028 | 1.050 | 2.365 |
|  | BAME | Reference | | | | | |
| Education Levels | Low | -0.890 | 0.2333 | 0.411 | 0.001 | 0.260 | 0.649 |
|  | Medium | -0.512 | 0.2640 | 0.599 | 0.052 | 0.357 | 1.005 |
|  | High | Reference | | | | | |
| Health Status | Good | 0.082 | 0.1964 | 1.086 | 0.675 | 0.739 | 1.596 |
|  | Poor | Reference | | | | | |
| Internet Use | Daily | 0.192 | 0.3204 | 1.212 | 0.549 | 0.647 | 2.271 |
|  | < daily | Reference | | | | | |
| Smartphone Owner | Yes | 1.132 | 0.3292 | 3.102 | 0.001 | 1.627 | 5.914 |
|  | No | Reference | | | | | |

| Q12 | What do you think the perception of artificial intelligence is in the media? | | | | | | |
| --- | --- | --- | --- | --- | --- | --- | --- |
|  |  | Coeff (B) | SE | Exp(B) | *p* value | 95% Confidence Interval | |
| Gender | Male | -0.009 | 0.2810 | 0.991 | 0.976 | 0.572 | 1.720 |
|  | Female | Reference | | | | | |
| Age | 16-30 | -0.804 | 0.4491 | 0.448 | 0.073 | 0.186 | 1.079 |
|  | 31-45 | -0.273 | 0.4470 | 0.761 | 0.542 | 0.317 | 1.829 |
|  | 46-64 | -0.344 | 0.4093 | 0.709 | 0.400 | 0.318 | 1.581 |
|  | 65+ | Reference | | | | | |
| Ethnicity | White | -0.597 | 0.3181 | 0.551 | 0.061 | 0.295 | 1.027 |
|  | BAME | Reference | | | | | |
| Education Levels | Low | -0.779 | 0.3590 | 0.459 | 0.030 | 0.227 | 0.927 |
|  | Medium | 0.140 | 0.3829 | 1.151 | 0.714 | 0.543 | 2.437 |
|  | High | Reference | | | | | |
| Health Status | Good | -0.032 | 0.2896 | 0.968 | 0.912 | 0.549 | 1.708 |
|  | Poor | Reference | | | | | |
| Internet Use | Daily | -0.016 | 0.5580 | 0.984 | 0.977 | 0.330 | 2.936 |
|  | < daily | Reference | | | | | |
| Smartphone Owner | Yes | 0.916 | 0.6105 | 2.499 | 0.134 | 0.755 | 8.268 |
|  | No | Reference | | | | | |

| Q13 | Do you trust artificial intelligence? | | | | | | |
| --- | --- | --- | --- | --- | --- | --- | --- |
|  |  | Coeff (B) | SE | Exp(B) | *p* value | 95% Confidence Interval | |
| Gender | Male | 0.386 | 0.2885 | 1.471 | 0.181 | 0.836 | 2.589 |
|  | Female | Reference | | | | | |
| Age | 16-30 | -0.185 | 0.4594 | 0.831 | 0.687 | 0.338 | 2.044 |
|  | 31-45 | -0.626 | 0.4622 | 0.535 | 0.176 | 0.216 | 1.323 |
|  | 46-64 | -0.146 | 0.4198 | 0.864 | 0.728 | 0.380 | 1.968 |
|  | 65+ | Reference | | | | | |
| Ethnicity | White | 0.684 | 0.3252 | 1.982 | 0.035 | 1.048 | 3.749 |
|  | BAME | Reference | | | | | |
| Education Levels | Low | -0.084 | 0.3615 | 0.920 | 0.817 | 0.453 | 1.868 |
|  | Medium | 0.364 | 0.3966 | 1.439 | 0.359 | 0.661 | 3.130 |
|  | High | Reference | | | | | |
| Health Status | Good | 0.175 | 0.2979 | 1.192 | 0.556 | 0.665 | 2.136 |
|  | Poor | Reference | | | | | |
| Internet Use | Daily | 0.006 | 0.5855 | 1.006 | 0.992 | 0.319 | 3.169 |
|  | < daily | Reference | | | | | |
| Smartphone Owner | Yes | 0.263 | 0.6093 | 1.300 | 0.666 | 0.394 | 4.292 |
|  | No | Reference | | | | | |

| Q14 | Do you think the benefits of using machine learning to analyse medical records to help diagnose patients outweigh the risks? | | | | | | |
| --- | --- | --- | --- | --- | --- | --- | --- |
|  |  | Coeff (B) | SE | Exp(B) | *p* value | 95% Confidence Interval | |
| Gender | Male | 0.690 | 0.3894 | 1.993 | 0.076 | 0.929 | 4.276 |
|  | Female | Reference | | | | | |
| Age | 16-30 | -0.412 | 0.5982 | 0.662 | 0.491 | 0.205 | 2.139 |
|  | 31-45 | -0.729 | 0.6150 | 0.482 | 0.236 | 0.145 | 1.611 |
|  | 46-64 | -0.673 | 0.5742 | 0.510 | 0.241 | 0.166 | 1.572 |
|  | 65+ | Reference | | | | | |
| Ethnicity | White | 0.259 | 0.4344 | 1.296 | 0.551 | 0.553 | 3.036 |
|  | BAME | Reference | | | | | |
| Education Levels | Low | 0.801 | 0.5167 | 2.227 | 0.121 | 0.809 | 6.132 |
|  | Medium | -0.302 | 0.5081 | 0.739 | 0.552 | 0.273 | 2.001 |
|  | High | Reference | | | | | |
| Health Status | Good | -0.569 | 0.3938 | 0.566 | 0.148 | 0.262 | 1.225 |
|  | Poor | Reference | | | | | |
| Internet Use | Daily | 0.915 | 0.7740 | 2.497 | 0.237 | 0.548 | 11.385 |
|  | < daily | Reference | | | | | |
| Smartphone Owner | Yes | -1.001 | 0.9564 | 0.367 | 0.295 | 0.056 | 2.394 |
|  | No | Reference | | | | | |

| Q15 | To what extent would you support the use of ML on the dataset to develop technology that could offer earlier diagnosis? | | | | | | |
| --- | --- | --- | --- | --- | --- | --- | --- |
|  |  | Coeff (B) | SE | Exp(B) | *p* value | 95% Confidence Interval | |
| Gender | Male | 0.216 | 0.3104 | 1.240 | 0.488 | 0.675 | 2.279 |
|  | Female | Reference | | | | | |
| Age | 16-30 | -1.240 | 0.5017 | 0.289 | 0.013 | 0.108 | 0.774 |
|  | 31-45 | -1.110 | 0.5058 | 0.327 | 0.027 | 0.121 | 0.880 |
|  | 46-64 | -0.530 | 0.4535 | 0.589 | 0.243 | 0.242 | 1.432 |
|  | 65+ | Reference | | | | | |
| Ethnicity | White | 0.911 | 0.3542 | 2.487 | 0.010 | 1.242 | 4.980 |
|  | BAME | Reference | | | | | |
| Education Levels | Low | -0.469 | 0.3907 | 0.626 | 0.230 | 0.291 | 1.346 |
|  | Medium | 0.811 | 0.4342 | 2.249 | 0.062 | 0.960 | 5.268 |
|  | High | Reference | | | | | |
| Health Status | Good | 0.196 | 0.3192 | 1.216 | 0.539 | 0.651 | 2.274 |
|  | Poor | Reference | | | | | |
| Internet Use | Daily | 1.067 | 0.6384 | 2.906 | 0.095 | 0.832 | 10.157 |
|  | < daily | Reference | | | | | |
| Smartphone Owner | Yes | 0.389 | 0.6515 | 1.475 | 0.550 | 0.411 | 5.291 |
|  | No | Reference | | | | | |

| Q16 | To what extent would you support the use of ML to interpret healthcare imaging? | | | | | | |
| --- | --- | --- | --- | --- | --- | --- | --- |
|  |  | Coeff (B) | SE | Exp(B) | *p* value | 95% Confidence Interval | |
| Gender | Male | 0.063 | 0.3044 | 1.065 | 0.835 | 0.587 | 1.935 |
|  | Female | Reference | | | | | |
| Age | 16-30 | -1.204 | 0.4941 | 0.300 | 0.015 | 0.114 | 0.790 |
|  | 31-45 | -0.1281 | 0.4995 | 0.278 | 0.010 | 0.104 | 0.739 |
|  | 46-64 | -0.319 | 0.4483 | 0.727 | 0.477 | 0.302 | 1.751 |
|  | 65+ | Reference | | | | | |
| Ethnicity | White | 0.591 | 0.3406 | 1.806 | 0.083 | 0.926 | 3.520 |
|  | BAME | Reference | | | | | |
| Education Levels | Low | -0.308 | 0.3803 | 0.735 | 0.417 | 0.349 | 1.548 |
|  | Medium | 1.090 | 0.4370 | 2.974 | 0.013 | 1.263 | 7.004 |
|  | High | Reference | | | | | |
| Health Status | Good | 0.335 | 0.3140 | 1.397 | 0.287 | 0.755 | 2.586 |
|  | Poor | Reference | | | | | |
| Internet Use | Daily | 0.351 | 0.6199 | 1.421 | 0.571 | 0.422 | 4.787 |
|  | < daily | Reference | | | | | |
| Smartphone Owner | Yes | 0.062 | 0.6468 | 1.063 | 0.924 | 0.299 | 3.778 |
|  | No | Reference | | | | | |
